# The influence of isolated and penta-hydrated Zn^2+^ on some of the intramolecular proton-transfer processes of thymine: a quantum chemical study †

**DOI:** 10.1039/c7ra13750h

**Published:** 2018-03-20

**Authors:** Dejie Li, Ying Han, Huijuan Li, Ping Zhang, Qi Kang, Zhihua Li, Dazhong Shen

**Affiliations:** College of Chemistry, Chemical Engineering and Materials Science, Collaborative Innovation Center of Functionalized Probes for Chemical Imaging in Universities of Shandong, Key Laboratory of Molecular and Nano Probes, Ministry of Education, Shandong Provincial Key Laboratory of Clean Production of Fine Chemicals, Shandong Normal University Jinan 250014 P. R. China; National Engineering Research Center for Colloidal Materials, School of Chemistry and Chemical Engineering, Shandong University Jinan 250100 P. R. China; College of Chemical and Environmental Engineering, Shandong University of Science and Technology Qingdao 266590 P. R. China

## Abstract

Zinc cation (Zn^2+^) plays an important role in the chemistry of DNA base pairs. In this work, the influence of isolated and penta-hydrated Zn^2+^ on some of the intramolecular proton-transfer processes of thymine (T) is investigated by the density functional theory method. It is shown that the calculated binding energies between Zn^2+^ and T are exothermic in vacuum. Compared to T, Zn^2+^ increases the stability of tautomer T′ by 28.7 kcal mol^−1^, promoting the intramolecular proton transfer of T. But in a micro-water environment, the attachment processes of Zn^2+^ to T hydrates, penta-hydrated Zn^2+^ to T, and penta-hydrated Zn^2+^ to T hydrates lead to the rearrangement of molecules and the redistribution of charges. The conventional T is still the most stable form and the influence of Zn^2+^ is much reduced and the proton transfer is thermodynamically unfavored. The detailed characterization is helpful to understand the genotoxicity of zinc ions.

## Abstract 2

The influence of isolated and penta-hydrated Zn^2+^ on some of the intramolecular proton-transfer processes of thymine is investigated by DFT modeling.

## Graphical abstract

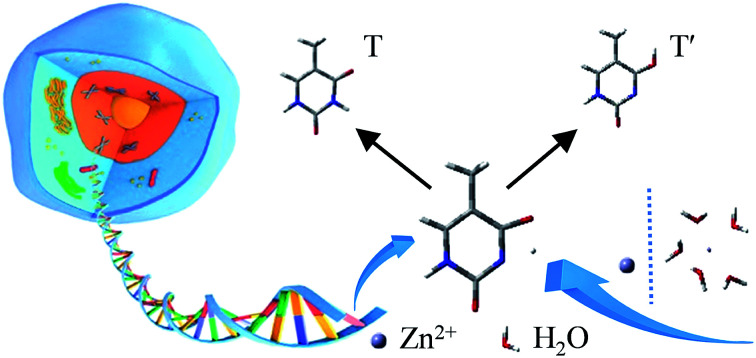

## Introduction

Many non-canonical tautomeric forms are generated by proton-transfer processes of nucleic acid (NA) bases in the chemical and biological sciences.^1^ It was demonstrated that the tautomeric equilibrium depends on the chemical environment, differing from crystalline state, aqueous or other solution and gas phase.^2–4^ Some factors such as excitation,^5^ metallic cation interaction,^6^*etc.*, are responsible for the tautomeric equilibrium between the canonical and non-canonical tautomeric forms. In addition, NA bases exposed to water exist in a number of biological structures. It is recognized that explicit water molecules act as both proton acceptor and donor, accelerating the tautomeric processes from canonical base to rare base.^7^

Thymine occurs naturally in DNA. For the adenine–thymine base pair, such a proton-transfer process would lead to thymine tautomerization and the mispairing of guanine and thymine tautomer.^8^ The biological importance of thymine has motivated a number of experimental^9–12^ and theoretical^13–15^ investigations. Previous experimental^16^ and computational^17^ studies of thymine were focused on both the canonical and rare tautomers. Suwaiyan^18^ studied the fluorescence of thymine tautomers at room temperature and investigated the relative stability of the canonical and rare tautomers. Hillier *et al.*^19^ analyzed the geometry of thymine and showed the difference in the order of stability of the tautomers. Meanwhile, the interaction of adenine–thymine with metal cations was also investigated.^20^

Metal cations play an important role in biological processes by forming non-covalent bonds, and acting as nonspecific binders as well.^21^ While alkali metals prefer to bind phosphate groups, transition metal cations such as zinc cation (Zn^2+^) are frequently localized near bases.^22^ As found in previous reports, the interactions between NA base pairs and metal cations have been extensively investigated theoretically^23^ and a good knowledge of the way they interact with DNA bases at the molecular level is of great importance. But the possible roles of metal cations, even “hydrated” metal cations, in the proton transfer of bases are still rarely reported. To understand the role of metal cations in the tautomeric equilibria of NA bases, it is necessary to carry out a detailed investigation of the intramolecular proton transfer of bases affected by metal cations.

It is well known that prototropy is a very fast and reversible process^24^ and is also very sensitive to experimental conditions.^25^ On the other hand, individual tautomers are very difficult to separate and detect.^26^ Consequently, normal experimental techniques, such as ultraviolet absorption spectrometry, infrared absorption spectrometry, Raman spectrometry, nuclear magnetic resonance, and mass spectrometry, have their own limits in the investigations of proton-transfer processes in complicated systems, especially related to tautomers.^27^ As an alternative strategy, quantum chemical methods such as the density functional theory (DFT) method have advantages in that they give the possibility of studying individual tautomers and intramolecular/intermolecular interactions.^24–26^ One can also model the tautomeric process and predict the microscopic parameters for isolated, micro-solvated and macro-solvated systems.^28^

In this work, Zn^2+^ and thymine are selected as models to study the intramolecular proton transfer (with/without water assistance) of thymine in the presence of Zn^2+^ (isolated or hydrated). Zn^2+^ is biologically important and may compete with other cations for complexation and water molecules could accelerate the tautomeric processes. A concern as to whether the interactions of isolated or hydrated Zn^2+^ are significantly large and could thus dramatically affect the tautomeric equilibria of thymine is discussed. Detailed structural parameters, energy changes, charge distributions and natural bond orbital (NBO) analysis were calculated by the DFT method.

## Computational methods

Theoretical studies of proton transfer in NA bases has been lasting for more than sixty years and various theories have already been proposed, such as semi-empirical molecular orbital theory,^29^*ab initio* molecular orbital theory^1,30–33^ and DFT.^27,28,34–36^ It is expected that both MP2 and B3LYP methods would give very similar results for the geometrical and vibrational features of bases,^37^ and DFT is an excellent compromise between computational cost and reasonable results.^38^ Previous research works^39^ employed the B3LYP/6-311++G** basis set to provide a much better energy description of the interaction between metal cations, NA bases, and micro-hydration bases. In this work, the optimization was performed by using a large basis set (6-311++G**) for a proper and accurate consideration. Energy profile, frequency calculation, as well as zero-point energy (ZPE) correction were performed at the same level of theory. All computed values were ZPE corrected unless otherwise noted.

The computed stationary points were characterized as minima or transition states by diagonalizing the Hessian matrix and analyzing the vibrational normal modes. In this way, the stationary points could be classified as minima if no imaginary frequencies were observed, or as transition states if only one imaginary frequency was obtained.^40^ The intrinsic reaction coordinate was followed with the DFT method to make sure that the transition state does connect the expected reactants and products. To investigate the interactions between metal cation and base, net atomic charges were obtained using the NBO analysis of Weinhold *et al.*^41^ Adiabatic potential energy surface (PES) changes along the N–H/O–H bond stretch were calculated using the optimization keyword opt = z-matrix, with the S action code in the additional input (N–H/O–H distance). Correspondingly, analysis of the charge distribution and molecular orbital information along with the changes of N–H/O–H distance was also made. All calculations were performed with the GAUSSIAN 03 ^42^ suite of packages.

## Results and discussion

### Stability of the Zn^2+^–thymine adducts

Schematic drawings of the optimized structures of 18 Zn^2+^–thymine adducts are shown in Fig. S1 in the (ESI†). Each of the adduct structures includes the tautomeric form of the base and the binding sites. These adducts are sequenced according to their relative energies. The results obtained by different methods are slightly different, but the variation trend is basically consistent, indicating such calculated results are still useful.

The canonical structure of thymine (T) with Zn^2+^ attached in Fig. S1 in ESI† represents the eighth or eleventh local minimum, which means that at least seven adducts of Zn^2+^–thymine are more stable. As can be seen in Fig. S1 in ESI,† T has only the unidentate binding sites available and the other seven adducts exhibit the N⋯Zn^2+^⋯O bidentate binding sites. In addition, all the stable adducts possess the carbonyl O⋯Zn^2+^⋯N binding motif. This motif is evidently more stable than the hydroxyl O⋯Zn^2+^⋯N ones. Of note is that the most favorable structure is composed of the rare tautomer (T′) and Zn^2+^, which is the first structure in Fig. S1 in ESI.† It is more stable than the metalated canonical form by as much as 23.2 kcal mol^−1^.

### Intramolecular proton transfer in Zn^2+^–thymine adducts

After establishing the order of the thermodynamic stability of the 18 Zn^2+^–thymine adducts, we focus on the tautomeric process and try our best to find the activation energy of the transition state. Note that not all of the tautomeric processes are discussed in this work, because the tautomers of thymine can be produced by proton transfer and internal rotation of the hydroxylic group. Thus, one tautomer can be converted to any other one, directly or indirectly. These transitions combined with water molecules that assist proton transfer can build up complicated networks between the tautomers.

**Fig. 1:**
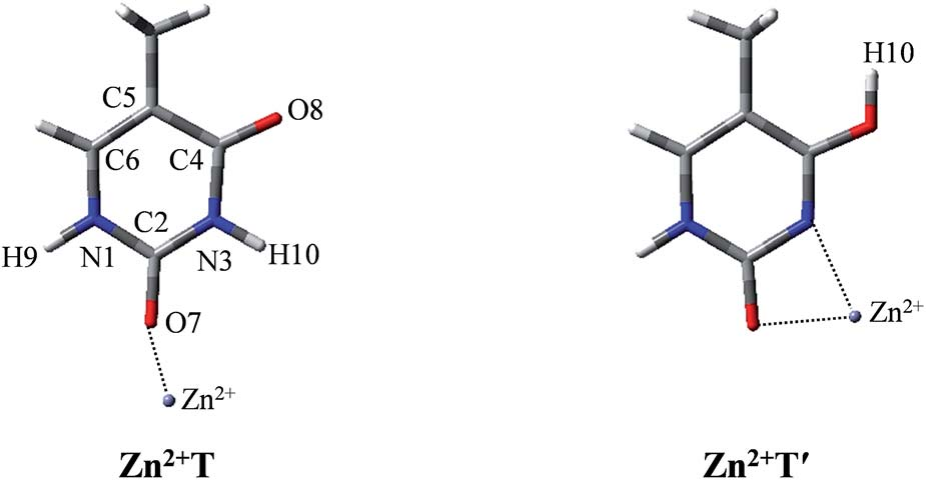
The molecular structures of the Zn^2+^–thymine adducts investigated. O, C, N atoms are shown in red, gray and blue colors, respectively.

T form can be found only in two of the Zn^2+^–thymine adducts shown in Fig. S1.† These two are different in the binding site of Zn^2+^ (see adducts 8 and 11 in Fig. S1†). In order to make a concise but in-depth discussion, the most likely pathway from one canonical structure to the favorable metalated rare tautomer, T and O7 attached Zn^2+^ adducts (Zn^2+^T) and the most favorable structure of the metalated rare tautomer (Zn^2+^T′), as shown in Fig. 1, are taken as the research objects for the intramolecular proton transfer of thymine. In addition, another possible tautomeric process from Zn^2+^T8 (number 8 represents tautomer 8 shown in Fig. S1†) to Zn^2+^T5 or Zn^2+^T4 is also investigated. Zn^2+^ is considered as an external influencing factor *via* the interactions occurring through another active site of T, that is, O8 position.

### Proton transfer in presence of Zn^2+^

For Zn^2+^T, the repulsion of Zn^2+^ with hydrogen of the amino group leads to the bending of the C2–O7–Zn^2+^ bond angle: the calculated bond angle of C2–O7–Zn^2+^ is 139.1° and bond distance of Zn^2+^–O7 is 1.904 Å. As listed in Table S1 in ESI,† net positive charge of Zn^2+^ is approximately equal to 1.3, indicating the charge transfer of almost one electron from T to Zn^2+^. This leads to the overall charge redistributions in the adduct, which enhances the electronegativity of the base center. As a result, the interaction of Zn^2+^ with T, which is primarily electrostatic, is reinforced during the charge redistribution.

**Fig. 2:**
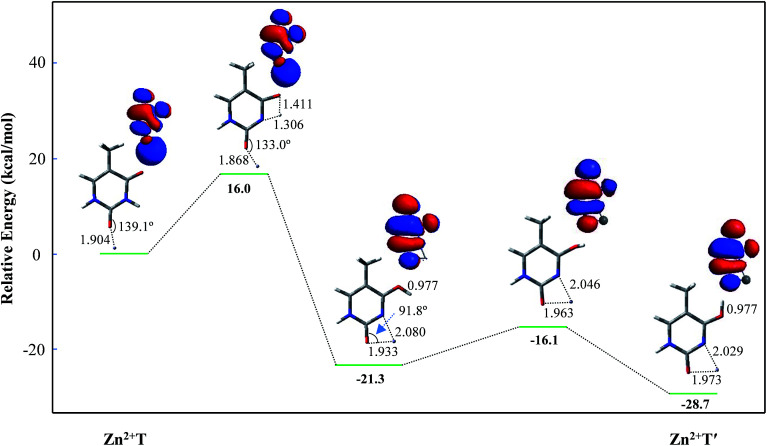
Energy profile of two-step proton transfer in the tautomeric process of Zn^2+^T → Zn^2+^T′. Sketches of the HOMOs are shown at top right of the structures and bond distances are in angstroms (Å).

Fig. 2 depicts the energy profile for Zn^2+^T → Zn^2+^T′, revealing that the tautomerization is a two-step process. The migration of H10 and metal cation produces a Zn^2+^–thymine bi-coordinated adduct (step 1), following by the internal rotation process of proton H10 (step 2).

With increasing of N3–H10 distance, the energy for the structure and charge distribution on Zn^2+^ are also increased. When the distances of N3–H10 and O8–H10 are 1.306 and 1.411 Å, respectively, the structure can be regarded a thermodynamic transition state. Single occupied molecular orbital (SOMO) remains a σ type bond from its initial σ bond and a dipole-bound contribution is dominant. Charge distribution on Zn atom varies from 1.333 to 1.437, and the value of activation energy is 16.0 kcal mol^−1^. Further on, with the N3–H10 distance increasing, *i.e.*, the O8–H10 distance decreasing, the structure energy decreased. The bond angle of C2–O7–Zn^2+^ varies from 133.0° to 91.8°. During this structural change, the relative energy is decreased. SOMO gradually becomes a π* bond from its initial σ* bond while the dipole-bound contribution is decayed. The stable structure is another form, stabilized by the four-membered chelate ring possessing bidentate coordination mode. The relative energy is −21.3 kcal mol^−1^, much lower than that of Zn^2+^T. Step 2 is the proton rotation process. The energy barrier of the internal rotation process is 5.2 kcal mol^−1^, which is obviously lower than that of the intramolecular proton-transfer process. Charge distribution on Zn^2+^ in Zn^2+^T′ increases to 1.650, near two atomic units of positive charge, confirming that the bidentate adduct Zn^2+^T′ adjusts the structure to a stable state with charge redistribution.

Since step 2 is of small energy barrier, the activation energy of the entire tautomeric process depends mainly on that in step 1. The activation energy (16.0 kcal mol^−1^) seems to make it difficult for H10 to transfer. However, the calculated binding energy between Zn^2+^ and T is −145.9 kcal mol^−1^ and such exothermic value could provide a driving force on thermodynamics for the tautomerization. Besides, Zn^2+^ increases the stability of proton-transferred structure Zn^2+^T′ by 28.7 kcal mol^−1^ compared to Zn^2+^T, indicating that the metal cation stabilizes greatly the non-classical form. Consequently, the tautomerization is favorable when the base is flooded with metal cations.^43^

As for the tautomeric process starting from Zn^2+^T8, a possible pathway shown in Fig. S2† reveals that Zn^2+^T8 → Zn^2+^T5 is a one-step process. Similar to Zn^2+^T → Zn^2+^T′, Zn^2+^T8 → Zn^2+^T4 is also a two-step process. Step 1 is the migration of H10 and metal cation producing Zn^2+^T5, followed by the internal rotation process of proton H10 to produce Zn^2+^T4 (step 2). The activation energies are 26.1 and 8.1 kcal mol^−1^ in steps 1 and 2, respectively. Both the energy barriers are more than those in Zn^2+^T → Zn^2+^T′ (Fig. 2), indicating that the tautomerization process is more prone to occur when Zn^2+^ occurs at the active site of O7. Therefore, the following investigations are focused on the adduct Zn^2+^T.

**Fig. 3:**
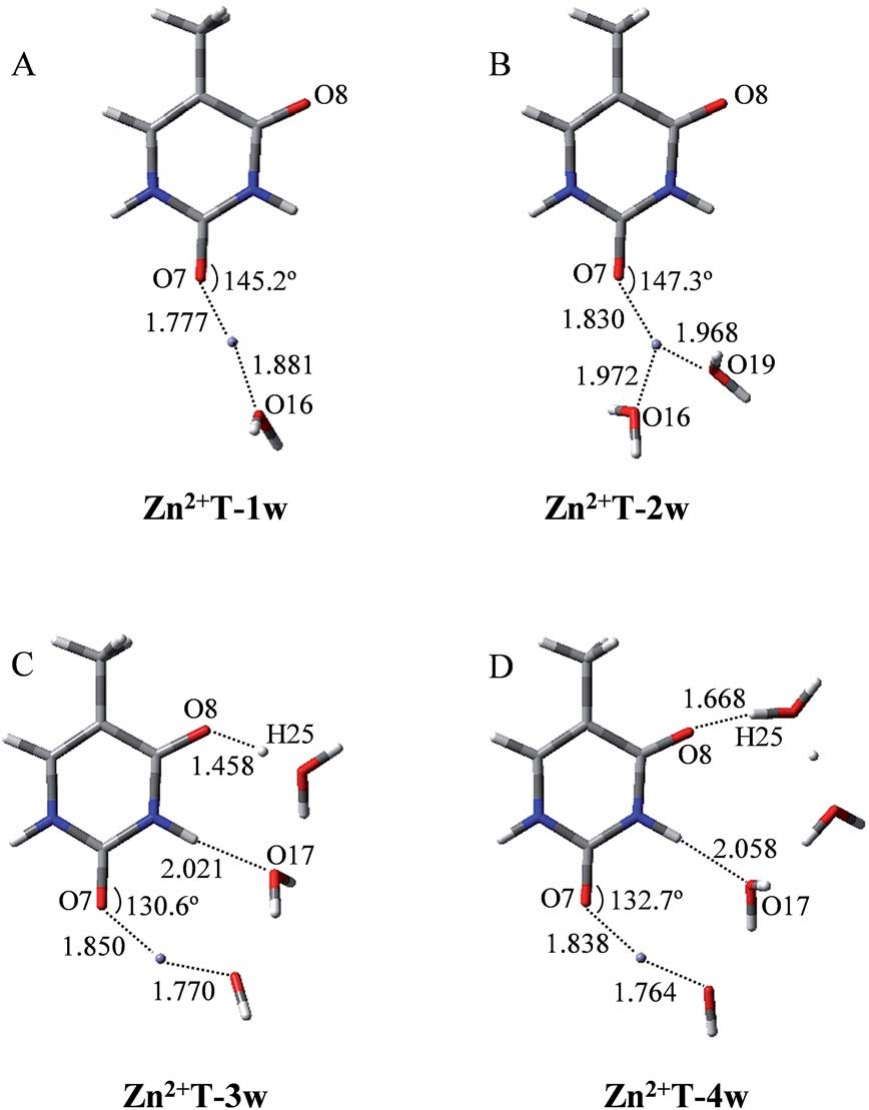
Optimized geometries of Zn^2+^ and hydrated T adducts. Bond distances in angstroms (Å).

It is worth noticing that the influence of water assisting proton transfer is also included, because water molecules can greatly decrease the activation energy in the base tautomerization. When water molecules act as both proton acceptor and donor, the energetically favored location is in the vicinity of O8–C4–N3–H10 region (Fig. 1). However, the optimized geometry in Fig. 3A shows that in Zn^2+^T-1w (the number represents water molecules attached and “w” is the abbreviation of water molecules), the six atoms (N1, C2, N3, O7, O16, Zn^2+^) are almost coplanar. The bond angle of Zn^2+^–O7–C2 is 145.2°, and bond lengths of Zn^2+^–O7 and Zn^2+^–O16 are 1.777 and 1.881 Å, respectively. Thus, the structure of mono-hydrated complex is obviously affected by Zn^2+^. The metal cation attached does not favor the tautomeric process.

As shown in Fig. 3B, the two water molecules coordinate to Zn^2+^ with nearly equivalent coordination bonds in Zn^2+^T-2w, with bond lengths of 1.972 and 1.968 Å for Zn^2+^–O16 and Zn^2+^–O19 respectively. Compared with the complex Zn^2+^T-1w, Zn^2+^–O7 bond has been further elongated by 0.053 Å, implying the gradual weakening of the interaction between Zn^2+^ and T. Optimization shows that the hydrogen atoms of the two water molecules are far from O7, the same as the water in Zn^2+^T-1w complex. Therefore, there is no path of proton transfer available for the tautomeric process when Zn^2+^ is attached.

How about the case of tri-hydrated Zn^2+^T-3w? As shown in Fig. 3C, the contact distance between Zn^2+^ and O7 is 1.850 Å, larger than those in Zn^2+^T-1w and Zn^2+^T-2w, suggesting the further weakening of the interactions of Zn^2+^ with T. The three water molecules and Zn^2+^ form a relatively long atom chain while there are only two water molecules forming a tautomeric path for proton transfer. However, in the water molecules adjacent to H10, both of the hydrogen atoms linked to O17 are pointing out of the tautomeric path, so the proton-transfer pathway is blocked.

**Fig. 4:**
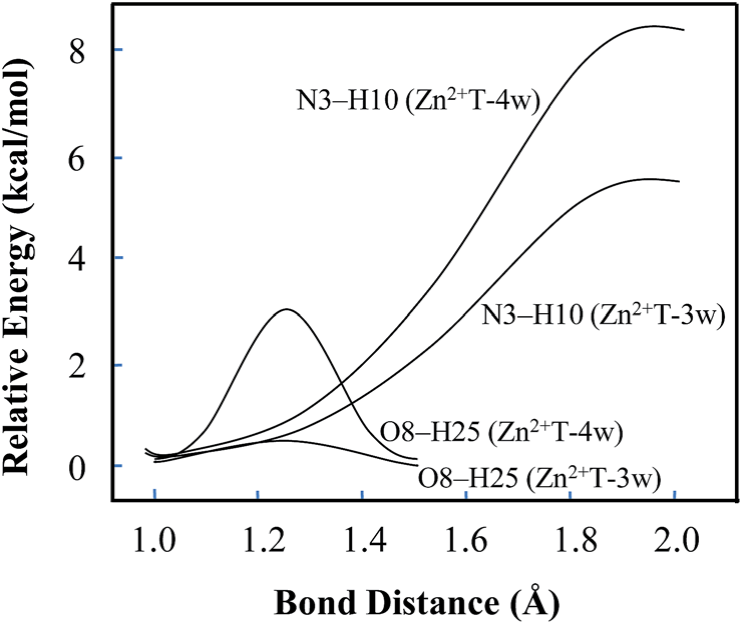
Potential energy changes along with the elongation of N3–H10 and O8–H25. The relative energy is based on the initial stable state of each structure.

In addition, several attempts to optimize the double proton-transferred structure failed. In order to observe the energy variation, the relationship of PES along N3–H10 and O8–H25 elongation were also optimized (H25 is the hydrogen of the water which is adjacent to O8). As depicted in Fig. 4, it is of interest that the energy profile of H25 dissociation is smooth, meaning it is barrierless (0.61 kcal mol^−1^); whereas the energy profile of H10 dissociation from N3 site is increased rapidly, implying an imaginary dissociation. Proton H10 is difficult to dissociate from T and transfer to the adjacent water, confirming that the proton transfer in Zn^2+^T-3w is impossible.

In Zn^2+^T-4w (Fig. 3D), the optimized structure shows the proximity of Zn^2+^ to capture one of the water molecules with strong positive charges, forming a linkage with two oxygens. The remaining three water molecules form an atomic chain with H10 and O8 such that protons appear to be transferable. However, due to the electrostatic effect, the captured water donates its hydrogen to the adjacent cluster of the other three water molecules. In this way, hydrogen in the water cluster is supersaturated and the charge distribution on the water cluster is 0.222 (Table S1 in ESI†). Hence, the electrostatic repulsion effect between H10 and the water cluster eventually hinders the transfer of protons. As H10 transfers from N3 to the adjacent water, the PES of Zn^2+^T-4w increases gradually without local energy minimum, resulting in an unavailable proton transfer even though the energy profile of H25 dissociation has a small barrier (3.3 kcal mol^−1^). In addition, optimization results show that in the water molecules adjacent to H10, both of the hydrogens are pointing out of the atom chain, so the proton-transfer pathway is also blocked, being similar to that in Zn^2+^T-3w. This further confirms that the tautomeric process will not occur in Zn^2+^T-4w.

### Proton transfer in presence of penta-hydrated Zn^2+^ (p-Zn^2+^)

**Fig. 5:**
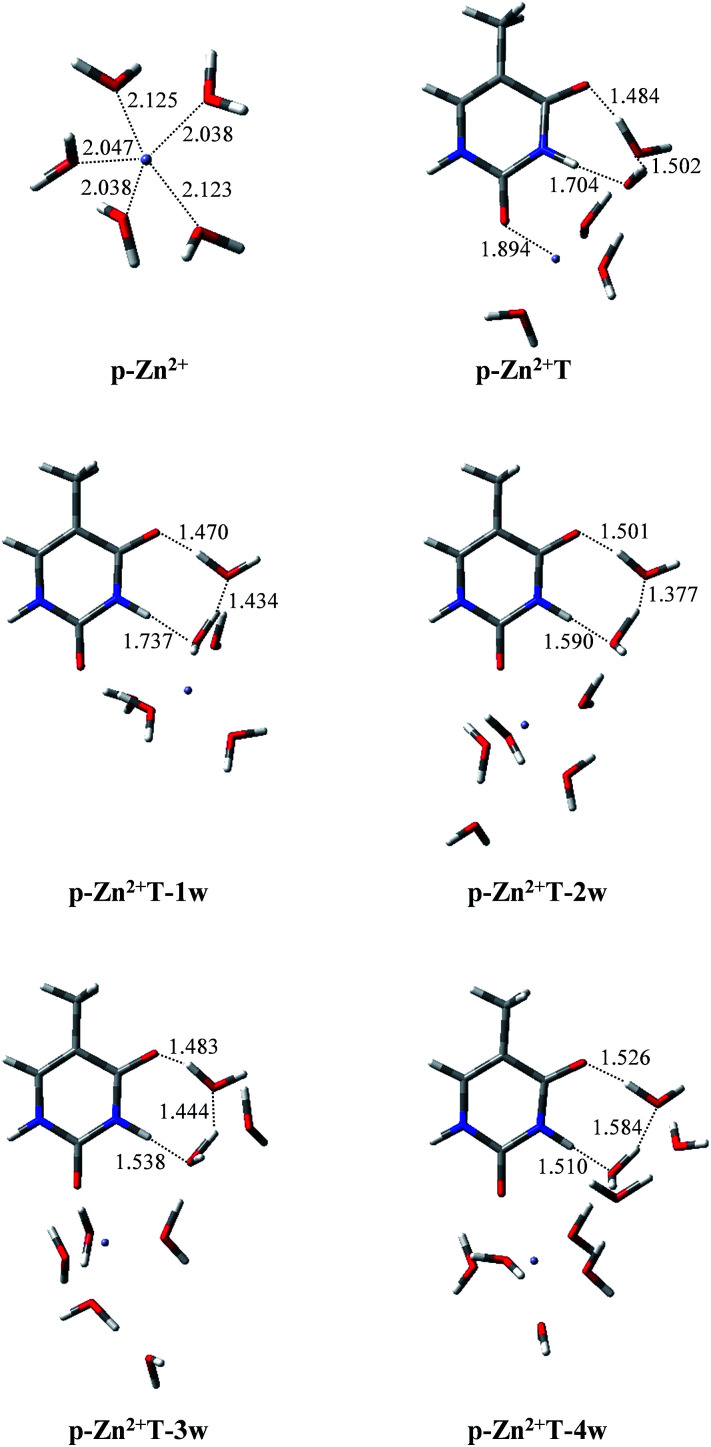
Optimized geometries of p-Zn^2+^, p-Zn^2+^T, p-Zn^2+^ and hydrated T adducts. Bond distances in angstroms (Å).

Infrared spectroscopy^44^ and theoretical studies^45^ of hydrated Zn^2+^ indicated that Zn^2+^ has a primary hydration sphere of five in the water environment. Therefore, investigations of the proton transfer of T and T hydrates affected by p-Zn^2+^ are also included in this work. As shown in Fig. 5, isolated p-Zn^2+^ has *C*_2V_ symmetry with four different Zn–O bond lengths. The average Zn–O bond length is 2.074 Å, which is in good agreement with previous result (2.077 Å).^44^ Charge distributions on the Zn^2+^ and the five water molecules are 1.714 and 0.286, respectively.

With p-Zn^2+^ attached (Fig. 5), the water molecules in the Zn^2+^ hydrate are rearranged and these adducts arrange two water molecules in the proton transfer area. We tried to find the proton-transferred stable structures in p-Zn^2+^T, p-Zn^2+^T-1w, p-Zn^2+^T-2w, p-Zn^2+^T-3w and p-Zn^2+^T-4w, but such structures do not exist. Optimized results indicate that all of the proton-transferred structures are not minima on the PES except the initial stable structures.

In T-2w, charge distribution on the two water molecules is just 0.024 in our calculated results. However, in the presence of p-Zn^2+^, more positive charges are redistributed on the two water molecules close to the area of proton transfer in the adducts. The charge distribution is 0.219, 0.215, 0.208, 0.198 and 0.176 in p-Zn^2+^T, p-Zn^2+^T-1w, p-Zn^2+^T-2w, p-Zn^2+^T-3w and p-Zn^2+^T-4w, respectively. The attachment of p-Zn^2+^ to T hydrates forms a positive electric field in the area of O8–C4–N3–H10 through water molecules, which impedes the transfer of proton H10. Therefore, the tautomeric process is thermodynamically unfavored in a water environment.

We are aware of the fact that the model selected for studying intramolecular proton-transfer processes of thymine under the influence of isolated and hydrated Zn^2+^ in this work has limitations. The results can represent only part of the proton-transfer processes and may not be transferred rigorously to solution.

## Conclusions

In this work, energy profiles and structural and electronic properties have been employed to characterize some of the tautomeric processes of thymine base by the DFT approach. With the number of surrounding water molecules increasing from 1 to 4, the process of T → T′ tautomerization affected by Zn^2+^ and p-Zn^2+^ is predicted.

(1) The order of the relative stability of the 18 thymine and Zn^2+^ adducts is estimated. Zn^2+^ association enhances the stabilization of the rare form of thymine. The adducts possessing the carbonyl O⋯Zn^2+^⋯N binding motif are more stable than the others.

(2) In the tautomeric process discussed in this work, the calculated binding energies between Zn^2+^ and T are highly exothermic, which is sufficient to satisfy the activation energy required for intramolecular proton transfer. Besides, Zn^2+^ increases the stability of proton-transferred structure compared to Zn^2+^T, which indicates that the metal cation can greatly stabilize the T′ form. Therefore, in a vacuum, Zn^2+^ can promote the intramolecular proton transfer of T.

(3) In a micro-water environment, the attachment processes of Zn^2+^ to T hydrates, p-Zn^2+^ to T, and p-Zn^2+^ to T hydrates will lead to the rearrangement of molecules and the redistribution of charges. The conventional thymine in keto form is still the most stable form and the proton transfer is thermodynamically unfavored. Water molecules play a very important role of buffering and the influence of Zn^2+^ on intramolecular proton-transfer processes of thymine is largely reduced.

## Conflicts of interest

There are no conflicts to declare.

## Supplementary Material

RA-008-C7RA13750H-s001
