# Supplementary material for: The influence of isolated and penta-hydrated Zn2+ on some of the intramolecular proton-transfer processes of thymine: a quantum chemical study
Source: RSC Adv. 2018 Mar 20;8(20):11021–6. doi: 10.1039/c7ra13750h (PMC9078977; doi:10.1039/c7ra13750h)
Supplement: RA-008-C7RA13750H-s001 [file RA-008-C7RA13750H-s001.pdf]

Electronic Supplementary Material (ESI) for RSC Advances.
This journal is © The Royal Society of Chemistry 2018

The influence of isolated and penta-hydrated Zn^{2+} on some of the intramolecular proton-transfer process of thymine: a quantum chemical study

Dejie Li, Ying Han, Huijuan Li, Ping Zhang, Qi Kang, Zhihua Li, Dazhong Shen

Corresponding author. Tel.: +86 0531 8618 0740; fax: +86 0531 8261 5258.

E-mail address: dzshen@sdnu.edu.cn (D. Z. Shen).

Fig. S1 Schematic drawings of the optimized structures of metalated thymine.

Fig. S2 Relative energy profile of the tautomeric process from Zn^{2+}T8 to Zn^{2+}T5 or Zn^{2+}T4.

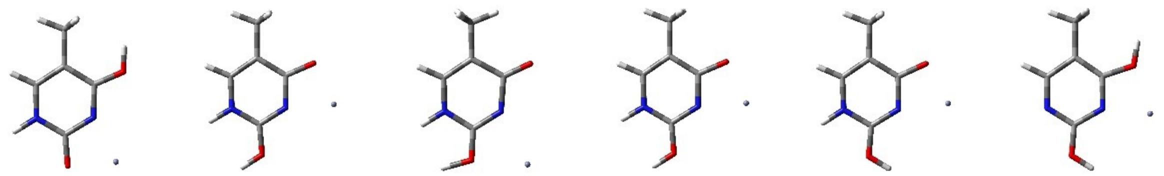

1 (-23.2) **2 (-21.6)** **3 (-20.7)** **4 (-19.6)** **5 (-18.4)** **6 (-18.2)**

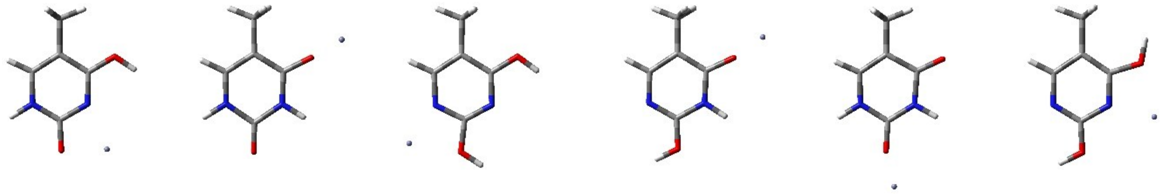

7 (-15.8) **8 (0)** **9 (3.3)** **10 (3.9)** **11 (5.5)** **12 (7.4)**

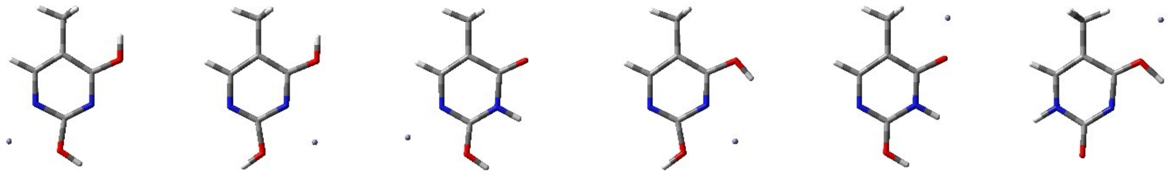

13 (7.5) **14 (8.4)** **15 (17.5)** **16 (17.6)** **17 (22.5)** **18 (41.4)**

Fig. S1 Schematic drawings of the optimized structures of metalated thymine. Each of the adduct structure covers the tautomeric form of the base and also information on the binding sites. They are sequenced in order of their relative energies according to the canonical structure of thymine with Zn^{2+} which attaches at O8 position. Energies are in kcal mol^{-1}.

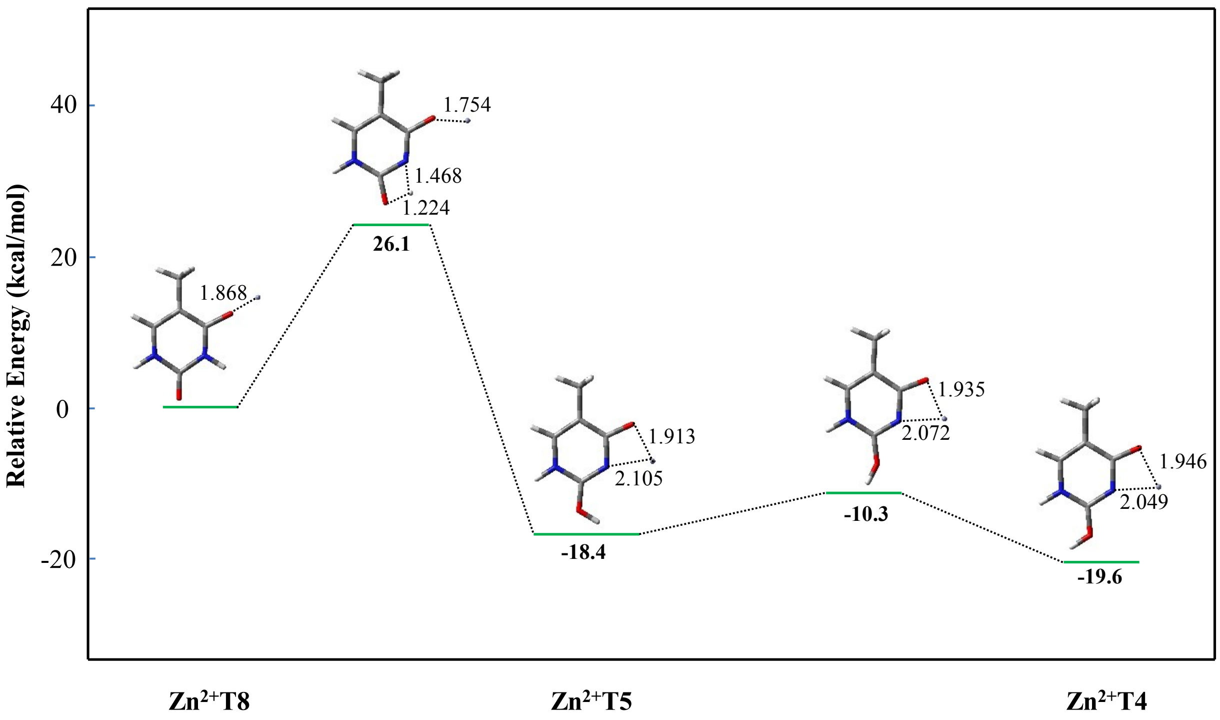

Fig. S2 Relative energy profile of the tautomeric process from Zn^{2+}T8 to Zn^{2+}T5 or Zn^{2+}T4. Zn^{2+}T8→Zn^{2+}T5 is a one step process and Zn^{2+}T8→Zn^{2+}T4 is a two steps process. Bond distances in angstrom.

Table S1 NPA Charge Distribution (au) on the Portion of Zn^{2+}T-nw

Water number	Zn^{2+}	T	water
0	1.333	0.667	–
1	1.697	0.195	0.108
2	1.726	0.129	0.145
3	1.646	0.175	0.179
4	1.624	0.154	0.222
